# Supplementary material for: Metabolic modelling and time-resolved mapping of glucose oxidative metabolism in the rat brain by indirect deuterium detection with 1H-FID-MRSI at 9.4 T
Source: MAGMA. 2026 May 27;39(3):381–93. doi: 10.1007/s10334-026-01363-6 (PMC13687330; doi:10.1007/s10334-026-01363-6)
Supplement: Supplementary file 1 [file 10334_2026_1363_MOESM1_ESM.docx]

**Supplementary Material**

Plasma glycemia - Group Average (n = 5)

mM

|  | Glycemia |  |
| --- | --- | --- |
| 0 | 5.96 | 0 |
| 6 | 16.36 | 0 |
| 26 | 20.72 | 0 |
| 46 | 22.04 | 0 |
| 66 | 23.34 | 0 |
| 86 | 22.62 | 0 |
| 106 | 21.84 | 0 |
| 126 | 20.1 | 0 |

*Figure S1: Group-averaged plasma glycemia measured along the 2H-Glc infusion protocol. Error bars represent the standard deviation or the measurements over the animal group.*

a)

b)

*Figure S2: ^2^H-Glx turnover curve along ^2^H-Glc infusion: scatter plot over the rats cohort for striatum (a) and hippocampus (b)*

*Figure S3: Representative LCModel fitting of pre- and post- 2H-Glc infusion (2 hours) ^1^H-[^2^H]-FID MRSI spectra, rephased via back-linear-prediction of missing FID points for the sake of visualization and normalized based on the NAA singlet peak. ^1^H Glx C4 signal reduction in the 2.3-2.4 ppm range, following metabolism of [6,6’-²H₂] glucose is clearly visible, while the rest of the spectral patterns remain stable.*
